# Diagnosis and treatment of advanced HER2-positive breast cancer in young pregnant female: A case report

**DOI:** 10.1097/MD.0000000000022929

**Published:** 2020-10-30

**Authors:** Tiantian Tang, Yueping Liu, Chao Yang, Li Ma

**Affiliations:** a Breast Center, the Fourth Hospital of Hebei Medical University; b Department of Pathology, the Fourth Hospital of Hebei Medical University, Shijiazhuang, China.

**Keywords:** case report, diagnosis, pregnancy-associated breast cancer (PABC), treatment

## Abstract

**Rationale:**

The incidence of pregnancy-associated breast cancer (PABC) is increasing nowadays, and its diagnosis and treatment remain complicated due to the consideration of the fetus. The available data on PABC are primarily derived from case reports since there are ethical restrictions on conducting randomized clinical trials. In the present work, we reported a case of the human epidermal growth factor receptor 2 (HER2)-positive PABC and described the diagnosis and treatment for such type of breast cancer.

**Patient concerns:**

A 27-year-old patient was admitted to our hospital with the complaints of right breast mass for 3 days, and she was a first-time pregnant woman with a single live intrauterine fetus at 26 + 3 weeks of gestation. Physical examination of the right breast revealed a palpable and hard mass with obscure boundaries (5.0 cm × 4.0 cm) in the upper outer quadrant. Significant axillary lymph nodes (2.0 cm) were also present.

**Diagnosis:**

PABC.

**Intervention:**

To protect the fetus, breast ultrasonography was used to test her breast mass, a core needle biopsy was adopted to confirm the diagnosis, and abdominal ultrasound and chest X-ray were used to evaluate the metastasis. The patient was scheduled for neoadjuvant therapy using bi-weekly pirarubicin in combination with cyclophosphamide (AC) without anti-HER2 therapy for consideration of the fetus's safety. After 4 cycles of AC, the patient delivered a healthy male infant. After the delivery, all the treatments were carried out according to the standard recommendation for HER2 + breast cancer as non-pregnant patients.

**Outcomes:**

After the surgery, the disease-free survival for the patient was 12 months until brain metastasis was diagnosed. She was still undergoing second-line anti-HER2 therapy and currently in a stable situation. Besides, the child was also healthy so far.

**Lessons:**

The methods for the diagnosis and treatment of PABC that result in teratogenesis should be avoided to protect the fetus. Mammogram and chest X-ray were safe approaches for the fetus. Moreover, chemotherapy-based on pirarubicin in combination with cyclophosphamide had no risk to the fetus.

## 1 Introduction

As a type of rare breast cancer, pregnancy-associated breast cancer (PABC) occurs in 1/10,000 to 1/3000 pregnancies,^[1–3]^ accounting for 1% to 5% in childbearing women.^[4]^ With the increase of reproductive age in recent years, the incidence of PABC is also increasing. PABC is more likely to have larger tumors, positive nodes, metastases, and vascular invasion due to the pregnancy-related changes in the breasts.^[5]^ Diagnosis and treatment of PABC are often unsatisfactory because of the consideration of the fetus, which also affects the outcome of PABC.^[6,7]^ The available data on PABC are primarily derived from case reports or case-control studies since there are ethical restrictions on conducting randomized clinical trials. Therefore, the diagnosis and treatment of PABC at different stages remain controversial.^[8,9]^ Multidisciplinary cooperation is essential for the management of PABC^[10,11]^. In the present case report, we aimed to evaluate the management of a patient with advanced human epidermal receptor-2 (HER2)-positive breast cancer during pregnancy and improve the prognosis of both the patient and fetus. Collectively, our findings provided a valuable clinical experience for the treatment of PABC.

## 2 Case presentation

**Figure 1:**
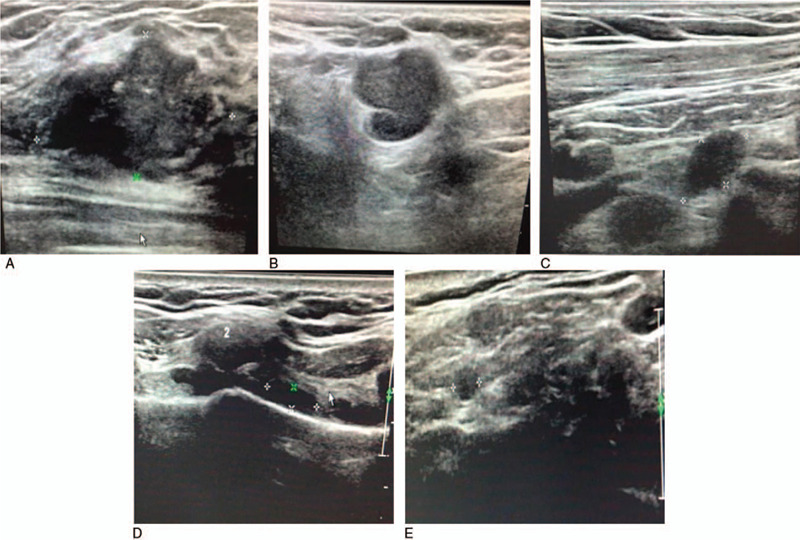
Imaging of right breast mass and right draining lymph node by ultrasound. (A) Right breast mass; (B) Right subaxillary lymph node; (C) Right subclavian lymph node; (D) Right internal mammary lymph node; (E) Right supraclavicular lymph node.

**Figure 2:**
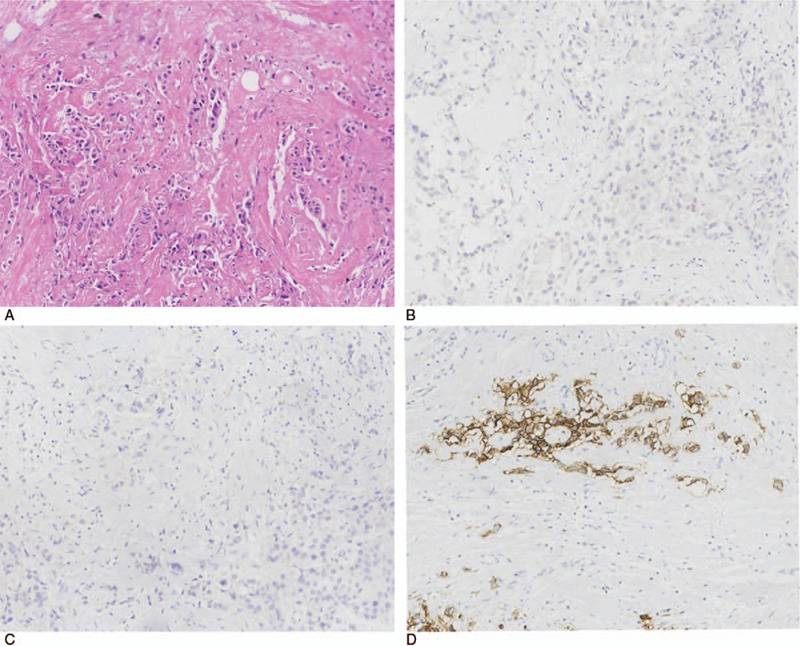
Core biopsy pathological results of right breast mass. (A) Original magnification × 200: HE staining showed invasive breast carcinoma in the right breast mass lesion; (B, C) Original magnification × 200: The tumor cells were negative for ER and progesterone receptors by IHC analysis in the breast mass; (D) Original magnification × 200: The tumor cells were positive for HER2 by IHC analysis in the breast mass. IHC = immunohistochemistry.

In Jun. 2017, a 27-year-old patient was admitted to our hospital with complaints of right breast mass for 3 days. The patient was a first-time pregnant woman with a single live intrauterine fetus at 26 + 3 weeks of gestation (second trimester). Physical examination of the right breast revealed a palpable and hard mass with obscure boundaries (5.0 cm × 4.0 cm) in the upper outer quadrant. Significant axillary lymph nodes (2.0 cm) were also present. Ultrasonography showed a mass of 4.4 cm × 3.8 cm × 2.3 cm on the right breast with enlarged lymph nodes in right subaxillary, internal mammary, subclavian and supraclavicular sections (Fig. 1). The patient did not take computed tomography (CT) because of the fetus. The abdominal ultrasound and chest X-ray showed no metastasis. A core needle biopsy of right breast mass was conducted, and histopathological examinations showed invasive breast carcinoma with lymphatic metastasis in the right subaxillary, subclavian, and supraclavicular lymph nodes. Immunohistochemistry on the right breast tumor showed negative staining for estrogen receptor and progesterone receptors but positive staining for the human epidermal receptor (HER2), and Ki-67 was positive for 20% (Fig. 2). The patient did not have a family history of breast cancer. The final diagnosis was PABC with T2N3M0-Stage IIIc, molecular typing HER2+.

The patient was scheduled for neoadjuvant therapy using bi-weekly pirarubicin in combination with cyclophosphamide (AC) without anti-HER2 therapy due to the consideration of the fetus's safety. The first chemotherapy cycle was started on Jul. 5, 2017 with intravenous (IV) injection of 90 mg pirarubicin (50 mg/m^2^) and 1100 mg cyclophosphamide on day 1 of the cycle. Similarly, her 2nd, 3rd, and 4th chemotherapy cycles containing AC regimen were given on Jul. 19, Aug. 4, and Aug. 18, 2018, respectively. An ultrasound antenatal scan with Doppler was performed at the end of every 2 cycles. On Sep. 13, 2017, after 4 cycles of AC, the ultrasound showed the presence of stable disease in breast mass (Table 1). There were no severe adverse reactions during chemotherapy, and fetal development was normal. The patient delivered a live male infant on Aug. 30, 2017. The birth weight of the infant was 2310 g, and his body length was 42 cm. Apgar score was also normal.

**Table 1:**
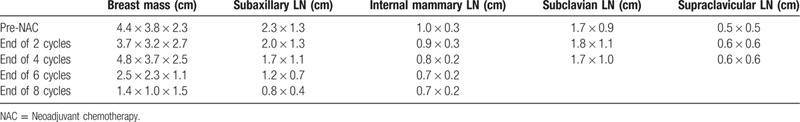
Evaluation of right breast mass and right draining lymph node by ultrasound during neoadjuvant chemotherapy.

**Figure 3:**
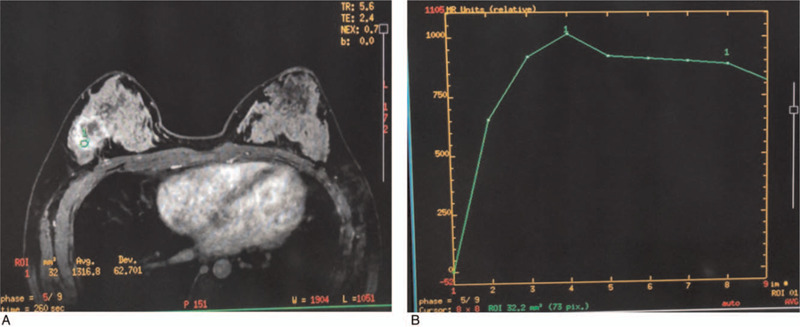
MRI imaging before the 5th cycle of neoadjuvant chemotherapy. (A) A 3.1-cm mass in right breast (BI-RADS 6); (B) time-signal curve demonstrated outflow type.

Magnetic resonance imaging (MRI) was performed before the 5th cycle, demonstrating a 3.1-cm mass with a 1.5-cm enlarged lymph node, BI-RADS 6 (Fig. 3). Brain, thoracic, and abdominal CT as well as bone scanning showed no signs of metastasis. The patient was switched to neoadjuvant therapy with TCbH every 21 days for 4 cycles. IV injection of paclitaxel (300 mg, 175 mg/m^2^) was given on day 1, carboplatin (300 mg) was given on day 1 and 2 (AUC = 6), and trastuzumab (8 mg/kg) was also intravenously injected for the first cycle on day 1, which was reduced to 6 mg/kg for the following cycles. The assessment was taken every 2 cycles.

**Figure 4:**
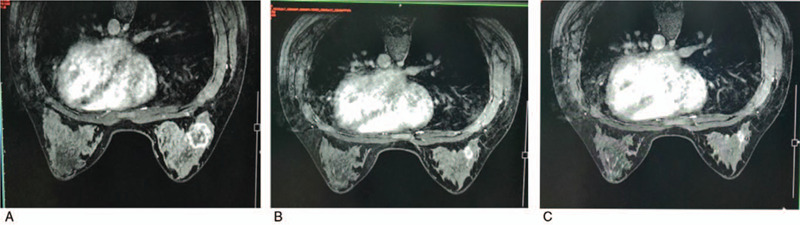
Evaluation of right breast mass by MRI during the neoadjuvant chemotherapy. (A) At the end of 4th cycle, a 3.1-cm breast mass was found; (B) at the 6th cycle, a 1.5-cm breast mass was found; (C) at the 8th cycle before operation, a 1.3-cm breast mass was found.

**Figure 5:**
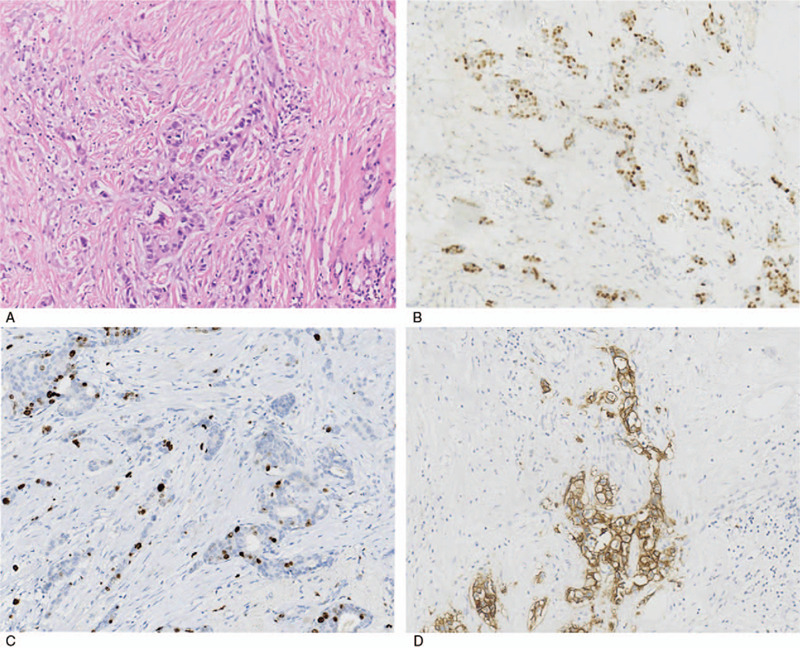
Postoperative pathological results of right breast mass. (A) Original magnification × 200: HE staining showed a little invasive ductal carcinoma with vascular invasion in the right breast mass lesion; (B, C) Original magnification × 200: The tumor cells were 40% and 20% positive for ER and progesterone receptors, respectively, by IHC analysis in the breast mass; (D) Original magnification × 200: The tumor cells were positive for human epidermal receptor-2by IHC analysis in the breast mass. IHC = immunohistochemistry.

At the end of the 8th cycle, the assessment was a partial response with MRI (Fig. 4). The patient underwent modified and extended radical mastectomy with right supraclavicular lymph node dissection on December 14, 2017. Postoperative histopathological examination revealed an invasive ductal carcinoma with vascular invasion (Fig. 5), with a size of 1.0 × 1.0 cm in the original tumor location (MP4). The axillary lymph node was 4/19 positive and 2/6 metastasis in the supraclavicular lymph nodes. Moreover, there was no lymph node metastasis in the internal mammary areas (0/1). Immunohistochemistry evaluation showed that the staining was ER-positive (40%) and progesterone receptors-positive (20%), and HER-2 staining was also positive (Fig. 5). Besides, tri-weekly TCbH was continuously given for two cycles as adjuvant chemotherapy. Radiotherapy was carried out to the remaining breast and drainage areas (46.8 Gy/26) as well as the internal mammary areas (55.9 Gy/26). Trastuzumab was given for 1 year, and endocrine therapy (goserelin 3.6 mg) was given every 28 days in combination with 2.5 mg of aromatase inhibitors every day. The patient was followed up in the outpatient service every 3 months.

The patient was hospitalized again in our center with the complaints of a headache in December 2018. Brain MRI showed multiple metastases in both sides of the cerebellum, and the largest mass had a maximum diameter of 3.5 cm. There was no other metastasis. The disease-free survival was 12 months.

**Figure 6:**
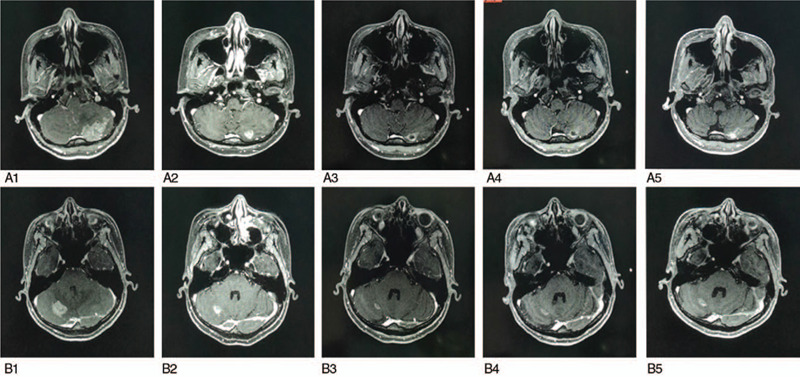
Evaluation of multiple metastases in both sides of cerebellum by MRI in 12-month follow-up. (A1–A5) Evaluation of metastatic lesion in left cerebellar hemisphere; (B1–B5) evaluation of metastatic lesion in right cerebellar hemisphere.

The patient received three-dimensional conformal radiation therapy (3DCRT) in combination with cyberknife therapy, and the systemic therapy was given based on pyrotinib and capecitabine every 21 days from December 2018 to Jun 2019. In Jun 2019, cerebrospinal fluid cytology showed tumor cells. Radioactive concentration could be seen in the 2nd and 6th ribs at the right by bone ECT. Progression-free survival for the patient was 6 months. Second-line treatment was intrathecal injection of methotrexate and dexamethasone plus IV zoledronic acid (4 mg) every month. The assessment was taken every 2 cycles by CT, and the treatment was continued until now (Fig. 6). The patient was currently in a stable condition, and her child was also healthy.

## 3 Discussion

PABC is defined as breast cancer occurring during pregnancy or within the first 1 to 2 years postpartum. The diagnosis and treatment of PABC present a challenge for patients, their relatives, and doctors. The pregnancy-related changes in the breast, such as increased breast density and nodularity, as well as complicated interpretation of clinical examination and breast imaging, make the diagnosis difficult. Additionally, changes in hormone levels during pregnancy stimulate breast carcinoma. Fetal development, whether and when to terminate the pregnancy are also focuses of PABC management. Some chemotherapeutic drugs associated with fetal malformations have made the treatment more complex.

When staging the PABC, the primary concern is the fetal radiation exposure. The threshold of maximum acceptable radiation for the fetus is 50 mGy. Congenital fetal loss, congenital anomalies, and intellectual effects occur when the radiation threshold reaches 60 to 200 mGy. Radiation exposure of mammogram and chest X-ray is 0.001 to 0.01 mGy and 0.0005 to 0.0001 mGy, respectively, and both of these examinations can be used during pregnancy when performed with proper fetal shielding.^[10]^ However, the increased density and nodularity of the breast during pregnancy may lower the sensitivity of mammography.^[12]^ Ultrasound is harmless to the fetus because there is no risk of any radiation exposure to the fetus, and ultrasonography is sensitive to dense breasts, therefore making it the best examination approach for the diagnosis of breast cancer during pregnancy. MRI is a reliable means to evaluate the therapeutic effect for patients with locally advanced breast cancer who require neoadjuvant therapy. However, little evidence of MRI is available in the application of PABC, and it is not used as a common method for the diagnosis of PABC due to its unknown effects on the fetus.^[8]^ When evaluating a patient's condition during pregnancy, we must consider the risks of ionizing radiation, bone scintigraphy, and CT, which are thus contraindicated.^[13]^ However, it is possible to use MRI without contrast.^[14]^ The gold standard for the diagnosis of PABC remains the histopathological diagnosis based on a core needle biopsy. Although it needs local infiltration anesthesia, such a little amount is harmless for the fetus.

It is particularly important to decide whether to terminate a pregnancy. There is no specific evidence that termination of pregnancy can improve the outcome of PABC patients. Patients should terminate the pregnancy when unfavorable prognosis and advanced-stage disease are detected in the first trimester.^[15]^ Different treatments have different effects on the fetus at different pregnancy stages. Therefore, it is essential to ensure the normal development of the fetus if the patient decides to continue the pregnancy.

Almost all known intravenous cancer therapies can penetrate the placental barrier and have fetal effects, such as fetal malformation, abortion, intrauterine growth retardation, and premature delivery.^[16]^ Chemotherapy should be avoided during the first trimester of pregnancy. Chemotherapy should be carefully chosen in the second and third trimesters. It is safe to choose chemotherapy after delivery. Breastfeeding is generally not recommended during chemotherapy because drugs are excreted in the patient's milk. Excretion depends on the capacity of the drug to bind to plasma proteins, as well as its liposolubility and ionization.^[17]^

Anti-HER2-based neoadjuvant therapy has become the standard treatment for HER2-positive breast cancer. Some anti-HER2 drugs, such as trastuzumab, are associated with oligohydramnios when administered for a long time during pregnancy.^[18]^ A study has shown that doxorubicin in combination with cyclophosphamide and 5-fluorouracil will not increase the pregnancy-related complications after 3 months of gestation, and anthracycline-based chemotherapy can be used with minimal risk to the fetus.^[19]^ Evidence shows that docetaxel/paclitaxel may have a shorter half-life in the plasma and a higher clearance during pregnancy. Therefore, it is a relatively safe choice for taxanes during the second and third trimesters of pregnancy.^[20]^ Based on the above-mentioned facts, anthracycline-based drugs are the first choice for PABC patients. Doses should not be reduced during pregnancy, which should be calculated based on the actual body weight of the patient. Pregnant patients should receive the last chemotherapy at least 4 weeks before the delivery to avoid high drug concentrations to infants at birth. In our present case, the patient was successfully treated with anthracycline in combination with cyclophosphamide for 4 cycles, she delivered a male infant during the 34th week of pregnancy with the normal birth weight and Apgar score, and no any abnormalities were observed. After the delivery, all the treatments were carried out according to the standard recommendation for HER2 + breast cancer as non-pregnant patients. Disease-free survival for the patient was 12 months until brain metastasis was diagnosed. Her metastasis might be attributed to the absence of trastuzumab in the initial treatment regimen.

## 4 Conclusions

Our current case report confirmed again that anti-HER2-based therapy was the standard regimen for PABC, highlighting the necessity and importance of anti-HER2 therapy for HER2 + breast cancer. The methods for the diagnosis and treatment of PABC that result in teratogenesis should be avoided to protect the fetus. Mammogram and chest X-ray were safe approaches for the fetus. Moreover, chemotherapy based on pirarubicin in combination with cyclophosphamide had no risk to the fetus.

## 5 Ethical statement

The study was approved by the Ethical Committees of the Fourth Hospital of Hebei Medical University. Information revealing the subject's identity was avoided. Written and signed informed consent was obtained from the patient to publish the case.

## Acknowledgments

The authors are grateful to all patients, clinicians, pathologists and statisticians who participated in this study

## Author contributions

**Conceptualization:** Tiantian Tang, Li Ma.

**Data curation:** Yueping Liu, Chao Yang.

**Formal analysis:** Tiantian Tang, Yueping Liu, Chao Yang, Li Ma.

**Funding acquisition:** Tiantian Tang, Li Ma.

**Investigation:** Tiantian Tang.

**Methodology:** Tiantian Tang.

**Project administration:** Li Ma.

**Resources:** Tiantian Tang.

**Software:** Yueping Liu.

**Validation:** Tiantian Tang, Yueping Liu, Chao Yang.

**Writing – original draft:** Tiantian Tang, Li Ma.

**Writing – review & editing:** Tiantian Tang, Li Ma.
